# IgA anti-CD74 autoantibodies are associated with treatment escalation in peripheral psoriatic arthritis

**DOI:** 10.3389/fimmu.2026.1821206

**Published:** 2026-05-18

**Authors:** Alaa Elsaghir, Luise Kuske, Katja Kniesch, Miriam Rabenow, Till Strowig, Torsten Witte

**Affiliations:** 1 Department of Rheumatology and Immunology, Hannover Medical School, Hannover, Germany; 2 Department of Microbiology & Immunology, Faculty of Pharmacy, Assiut University, Assiut, Egypt; 3 Helmholtz Centre for Infection Research, Braunschweig, Germany; 4 Center for Individualized Infection Medicine, a joint venture of Hannover Medical School and the Helmholtz Center for Infection Research, Hannover, Germany

**Keywords:** psoriatic arthritis, peripheral psoriatic arthritis, IgA anti-CD74, biomarker, treatment escalation, bDMARDs, csDMARDs

## Abstract

**Background:**

Psoriatic arthritis (PsA) often requires escalation from conventional synthetic disease-modifying antirheumatic drugs (csDMARDs) to biologic therapy (bDMARDs), yet biomarkers guiding treatment decisions remain limited. Anti-CD74 autoantibodies have shown diagnostic potential in axial spondyloarthritis, but their relevance in PsA is insufficiently characterized.

**Objective:**

To evaluate whether IgA anti-CD74 levels are associated with treatment escalation in peripheral PsA (pPsA) and to characterize their relationships with clinical and immunological parameters.

**Methods:**

Serum samples from 171 PsA (127 pPsA, 44 axial PsA [axPsA]), 43 non-rheumatic disease controls (NRD), and 43 rheumatoid arthritis (RA) patients were analyzed. IgA anti-CD74 levels were measured by enzyme-linked immunosorbent assay (ELISA). Patients were stratified by disease duration and treatment exposure. Correlation analyses, receiver operating characteristic (ROC) curves, and logistic regression models were performed. A prospective subgroup of 53 early pPsA patients was followed to assess treatment initiation.

**Results:**

IgA anti-CD74 levels were elevated in PsA compared with NRD controls (median 13 vs. 6 U/mL, p < 0.0001), with similar levels in pPsA and axPsA and comparable values in RA, indicating an association with inflammatory disease rather than disease specificity. Anti-CD74 positivity (>15 U/mL) was observed in 31.5% of pPsA and 43.2% of axPsA versus 2.3% of NRD, independent of disease duration. Anti-CD74 levels were associated with treatment escalation in pPsA, with higher levels in bDMARD-treated patients (median 13.0 vs. 11.0 U/mL, p = 0.04). In multivariate analyses, anti-CD74 was independently associated with csDMARD (OR 1.113, p = 0.022) and bDMARD use (OR 1.052, p = 0.02). After false discovery rate (FDR) correction, anti-CD74 remained associated with serum IgA (q = 0.0008) and weakly with IgG (q = 0.0250), but not with C-reactive protein (CRP) or age. Longitudinal associations were not significant after FDR correction (csDMARD initiation: p = 0.047, q = 0.094; bDMARD initiation: p = 0.19, q = 0.1866), indicating these findings are exploratory.

**Conclusion:**

IgA anti-CD74 levels are elevated in PsA and appear to reflect immunological activity not captured by CRP. Their independent association with treatment escalation in pPsA supports further evaluation as a biomarker candidate, although findings remain exploratory and require validation in larger longitudinal cohorts.

## 1 Introduction

Psoriatic arthritis (PsA) is a chronic, immune-mediated inflammatory disorder characterized by diverse clinical manifestations across multiple disease domains, including peripheral arthritis, enthesitis, dactylitis, psoriasis, nail disorders, and, in a subset of patients, axial involvement (1). PsA exhibits clinical heterogeneity, characterized by diverse disease trajectories and treatment responses, frequently necessitating escalation to conventional synthetic disease-modifying antirheumatic drugs (csDMARDs), biologic treatments (bDMARDs), or JAK inhibitors (targeted synthetic (ts)DMARDs) (2, 3) Despite the increase in therapeutic options, approximately 50-60% of patients respond well to the first DMARD therapy, underscoring the need for predictive biomarkers to guide individualized treatment decisions (4, 5).

Treatment decisions in PsA are largely guided by clinical assessment and conventional inflammatory markers such as C-reactive protein (CRP), which do not fully capture disease heterogeneity or underlying immunopathology (6). Consequently, there is growing interest in mechanistically informative biomarkers that reflect disease-relevant immune pathways and may improve patient stratification and prediction of treatment requirements. Such biomarkers could provide information beyond general inflammation and help identify patients at risk of requiring early treatment escalation.

Anti-CD74 autoantibodies have emerged as potential biomarkers in axial spondyloarthritis (axSpA). Several studies have demonstrated elevated levels of IgA anti-CD74 antibodies in axSpA, with reported diagnostic utility and high specificity in selected patient populations (7–12). A systematic review and meta-analysis confirmed that both IgA and IgG anti-CD74 antibodies are significantly increased in axSpA compared with controls (10). In addition, longitudinal data suggest that anti-CD74 IgA levels may be associated with structural disease progression (11, 12). However, these findings are not entirely consistent, and anti-CD74 antibodies show limited correlation with disease activity measures and do not reliably distinguish axial disease from peripheral psoriatic arthritis (pPsA) (13).

Importantly, the role of anti-CD74 in PsA, particularly in peripheral disease phenotypes, remains insufficiently characterized. While previous studies have primarily focused on diagnostic performance in axSpA, their potential relevance as biomarkers reflecting disease-related immune mechanisms and treatment requirements in PsA has not been systematically evaluated.

CD74 is a key component of the major histocompatibility complex class II (MHC II) pathway, involved in antigen presentation and macrophage migration inhibitory factor (MIF)-mediated signaling, both of which are implicated in chronic inflammatory responses (14, 15). The predominance of the IgA isotype in anti-CD74 responses further suggests a potential link to mucosal immune activation, particularly along the gut–joint axis, which has been increasingly implicated in the pathogenesis of spondyloarthritis and psoriatic disease (16–18).

Taken together, these observations support the hypothesis that anti-CD74 IgA may reflect a distinct immunological axis not captured by conventional inflammatory markers. However, whether this biomarker is clinically relevant in PsA, particularly in relation to treatment escalation and disease stratification, remains unknown. Accordingly, the primary objective of this study was to evaluate the association between serum IgA anti-CD74 levels and treatment escalation in patients with pPsA. Secondary objectives were to characterize anti-CD74 levels across disease subgroups and disease duration, and to assess their relationship with clinical and immunological parameters, including CRP and age. Exploratory analyses were conducted to examine potential modifiers, including BMI, psoriasis outcomes, and early treatment initiation.

By integrating cross-sectional and prospective data, this study aims to determine whether anti-CD74 represents a clinically relevant biomarker associated with treatment patterns and to provide insight into its potential role in guiding disease stratification in PsA.

## 2 Materials and methods

### 2.1 Study population

Serum samples were collected from 171 patients clinically diagnosed with PsA by rheumatologists at the Department of Rheumatology and Immunology, Hannover Medical School, Germany. Between 2018 and 2024, all patients referred to the department for suspicion of PsA were invited to participate in the study. In addition, individuals presenting with musculoskeletal pain during the same period, but in whom no inflammatory rheumatic disease was diagnosed, were recruited as controls.

#### 2.1.1 PsA subgroup without axial involvement [termed peripheral PsA (pPsA)]

A total of 127 patients presented without clinical inflammatory back pain or radiographic evidence of axial involvement (pPsA). Within this group, 53 patients had a disease duration ≤ 1 year since the onset of arthritis, and 74 patients had a disease duration > 1 year.

#### 2.1.2 Axial PsA subgroup

In addition, 44 PsA patients showed concomitant axial involvement (axPsA), defined by clinical features (such as inflammatory back pain) and/or imaging findings indicative of axial involvement, consistent with previously established definitions (19, 20). This definition was applied to ensure alignment with current literature and improve the accuracy of axial disease classification. Six patients had a disease duration ≤ 1 year, while 38 patients had a disease duration > 1 year.

Additionally, 43 participants with no inflammatory rheumatic diseases (NRD) served as controls. These NRD controls were referred due to musculoskeletal pain, but after careful evaluation had no clinical or laboratory evidence of any inflammatory rheumatic condition.

Moreover, a subgroup of patients with rheumatoid arthritis (RA, n = 43) was included as an inflammatory disease control group to enable comparative assessment of IgA anti-CD74 levels across inflammatory and non-inflammatory musculoskeletal conditions encountered in routine clinical practice.

#### 2.1.3 Prospective follow-up

The subgroup of 53 patients with early pPsA (disease duration ≤ 1 year) was prospectively followed to assess treatment outcomes. During follow-up, initiation of csDMARDs and bDMARDs was recorded as binary variables (1/0). Baseline anti-CD74 levels were used for both cross-sectional and longitudinal analyses.

Anti-CD74 IgA levels were assessed only at baseline and were used to evaluate their association with subsequent treatment escalation. Repeated anti-CD74 measurements were not available.

Additional clinical data, including height, weight, and psoriasis status during follow-up, were available for the early pPsA subgroup, Body mass index (BMI) was calculated, and psoriasis outcomes following methotrexate therapy were categorized descriptively. These variables were used in exploratory analyses to evaluate potential associations with baseline IgA anti-CD74 levels and treatment patterns.

For BMI-related exploratory analyses, csDMARD and bDMARD exposure were evaluated separately as binary variables (1/0), irrespective of concomitant therapy. This classification was applied to capture major therapeutic categories relevant to clinical decision-making, while acknowledging that treatment strategies in PsA are heterogeneous and may involve combination regimens.

The study involving human participants obtained permission from the local Ethics Committee of Hannover Medical School (approval number 5582). All participants provided written informed consent before their participation in the study, in accordance with the Declaration of Helsinki.

### 2.2 Laboratory data

Clinical and laboratory variables, including age, sex, disease duration, axial involvement, arthritis, enthesitis, dactylitis, bursitis, inflammatory bowel disease (IBD), uveitis, current smoking status (at the time of visit), C-reactive protein (CRP), HLA-B27 status, and treatment information, were collected by the treating rheumatologist and recorded in the study database.

Serum IgA antibodies against CD74 were measured using a commercially available ELISA kit (SpADetect, Aesku. Diagnostics, Wendelsheim, Germany), employing full-length recombinant human CD74 produced in HEK293 cells as the antigen.

A threshold of >15 U/mL was defined to indicate anti-CD74 positivity based on receiver operating characteristic (ROC) curve analysis performed in our cohort. This cutoff was selected to prioritize high specificity for distinguishing PsA from non-inflammatory musculoskeletal conditions, thereby minimizing false-positive classification in a clinically heterogeneous population.

Although the manufacturer defines values ≤20 U/mL as within the normal range, lower thresholds have been reported in previous studies of spondyloarthritis and were also supported by the optimal discrimination observed in our dataset. Accordingly, the selected cutoff represents a data-driven approach, aligned with existing literature, and optimized for the intended clinical application rather than relying solely on manufacturer-defined reference values (21, 22).

### 2.3 Statistical analysis

Statistical analyses were performed using GraphPad Prism version 8.3 (GraphPad Software Inc., San Diego, CA, USA). Categorical variables are presented as counts and percentages. Continuous variables are expressed as median and interquartile range (IQR) for non-normally distributed data, or as mean ± standard deviation (SD) for normally distributed data. Normality was assessed using the Shapiro–Wilk test.

Comparisons between two groups were performed using the Mann–Whitney U test for non-parametric data. Comparisons across multiple groups were conducted using the Kruskal–Wallis test followed by Dunn’s *post hoc* test for multiple comparisons. Categorical variables were analyzed using Fisher’s exact test. Correlations between continuous variables were assessed using Spearman’s rank correlation coefficient.

Receiver operating characteristic (ROC) curve analysis was used to evaluate the discriminative performance of anti-CD74 and to determine the optimal cutoff value. The area under the curve (AUC), 95% confidence interval (CI), sensitivity, specificity, and positive and negative likelihood ratios were calculated.

Univariate and multivariate logistic regression analyses were performed to assess the association between anti-CD74 levels and treatment categories.

To align the statistical approach with the clinical and mechanistic objectives of the study, a hierarchical analytical framework was predefined. The primary objective was to evaluate whether IgA anti-CD74 levels are associated with treatment escalation in pPsA, assessed by csDMARD and bDMARD use.

Secondary analyses were designed to contextualize this association by examining whether anti-CD74 levels differ across disease groups and disease duration, and whether they are associated with conventional clinical and inflammatory parameters, including CRP, age, and other clinical features. These analyses aimed to determine whether anti-CD74 reflects disease-related immunological processes independent of traditional markers.

Exploratory analyses were conducted to further investigate potential modifiers of anti-CD74 levels and treatment patterns, including BMI, psoriasis outcomes following methotrexate therapy, and treatment initiation in the early pPsA subgroup. These analyses were hypothesis-generating and intended to provide additional clinical context.

Given the number of statistical tests performed, particularly within exploratory analyses, a global correction for multiple testing was not applied. Instead, false discovery rate (FDR) correction using the Benjamini–Hochberg method was applied to predefined families of related exploratory analyses (including correlation analyses, BMI-related analyses, and early longitudinal subgroup analyses) to assess the robustness of these findings. Primary hypothesis-driven analyses, regression models, and *post hoc* group comparisons already adjusted for multiple testing (e.g., Dunn’s test) were not subjected to additional correction.

A p-value < 0.05 was considered statistically significant. For analyses in which FDR correction was applied, adjusted p-values (q-values) < 0.05 were considered statistically significant.

## 3 Results

### 3.1 Patient demographics and clinical characteristics

Patients with pPsA predominantly exhibited peripheral manifestations such as arthritis and enthesitis, whereas these features were less frequent in axPsA. CRP elevation was more common in both PsA groups than in NRD controls, consistent with an inflammatory phenotype. Smoking was more frequent among NRD controls.

RA patients were older than pPsA patients and showed higher RF and ACPA positivity, as expected. CRP elevation was also more frequent in RA, confirming the inflammatory nature of this comparator group (**Table 1**).

**Table 1:** Demographic and disease characteristics at collection date for patients and control group.

Variables		pPsA, (*n=127*)	axPsA, (*n=44*)	NRD, (*n=43*)	RA, (*n=43*)		Missing data (n)			*p-*value (NRD vs pPsA)	*p-*value (NRD vs axPsA)	*p-*value (RA vs pPsA)
						pPsA (n)	axPsA (n)	NRD (n)	RA (n)			
Gender (n, %)	Male	51 (40.16)	15 (34.09)	11 (25.58)	11 (25.58)	–	–	–	–	0.1	0.48	0.1
	female	76 (59.84)	29 (65.91)	32 (74.42)	32 (74.42)							
Age (mean, SD)		51.6 (15.7)	51.8 (11.5)	53 (15.1)	60.30 (12.75)	–	–	–	–	0.15	0.7	0.0012
Axial involvement (n, %)		0 (0)	44 (100)	NA	1 (2.33)	–	–	–	–	NA	NA	NA
Arthritis (n, %)		113 (88.97)	30 (68.18)	NA	42 (97.67)	–	–	–	–	NA	NA	0.12
Enthesitis (n, %)		57 (44.88)	13 (29.55)	NA	0 (0)	–	–	–	–	NA	NA	NA
Dactylitis (n, %)		38 (29.92)	10 (22.73)	NA	0 (0)	–	–	–		NA	NA	NA
Uveitis/Iritis (n, %)		2 (1.57)	2 (4.55)	NA	0 (0)	–	–	–	–	NA	NA	NA
IBD (n, %)		2 (1.57)	1 (2.27)	NA	0 (0)	–	–	–	–	NA	NA	NA
Bursitis (n, %)		9 (7.08)	0 (0)	NA	0 (0)	–	–	–	–	NA	NA	NA
Smoking (n, %)		11 (8.8)	5 (11.36)	24 (55.8)	7 (16.28)	2	–	–	–	<0.0001	<0.0001	0.3
CRP>5mg/l (n, %)		28 (22.22)	11 (25)	6 (13.95)	24 (55.81)	1	–	–	–	0.278	0.28	<0.0001
HLA-B27 (n, %)		9 (10.59)	7 (20)	2 (20)	NA	42	9	33	–	0.33	>0.9999	NA
RF (n, %)		3 (2.36)	1 (2.27)	6 (13.95)	16 (37.21)	–	–	–	–	0.009	0.058	<0.0001
ACPA (n, %)		0 (0)	0 (0)	NA	15 (34.88)	–	1	–	–	NA	NA	NA
NSAIDs (n, %)		32 (25.19)	21 (47.73)	NA	8 (18.60)	3	–	–	–	NA	NA	NA
OGCs (n, %)		21 (16.54)	8 (18.18)	NA	30 (69.77)	1	–	–	–	NA	NA	NA
csDMARDs use (n, %)		106 (83.46)	35 (79.55)	NA	42 (97.67)	1	–	–	–	NA	NA	NA
bDMARDs use (n, %)		71 (55.9)	32 (72.73)	NA	28 (65.12)	1	–	–	–	NA	NA	NA

NA, means not applicable; pPsA, Psoriatic Arthritis without axial involvement (peripheral PsA); axPsA, Psoriatic Arthritis with axial involvement; RA, rheumatoid arthritis; IBD, inflammatory bowel disease; CRP, C-reactive protein; HLA-B27, Human Leucocyte Antigen B27; RF, rheumatoid factor; ACPA, anti-citrullinated protein antibodies; NSAID, nonsteroidal anti-inflammatory drug; OGCs, oral glucocorticoids; csDMARDs, conventional synthetic Disease-Modifying Antirheumatic Drugs; bDMARDs, biologic Disease-Modifying Antirheumatic Drugs. A p-value < 0.05 was considered statistically significant.

### 3.2 Anti-CD74 levels in PsA and disease discrimination

IgA anti-CD74 levels were significantly elevated in PsA compared to NRD controls across all disease subsets. Median (IQR) levels were 13 (8–19) U/mL in total PsA, 12 (7–19) U/mL in pPsA, 15 (12–19.75) U/mL in axPsA, and 6 (4–9) U/mL in NRD (all p < 0.0001 vs NRD). No significant differences were observed between pPsA and axPsA, indicating comparable antibody expression across clinical phenotypes.

There were no statistically significant differences in anti-CD74 levels between the RA and any PsA subgroups (all p > 0.05). This finding indicates that elevated anti-CD74 levels are not disease-specific but reflect a shared feature of inflammatory rheumatic conditions.

Stratification by disease duration showed that anti-CD74 levels were elevated in both early (≤1 year) and late (>1 year) pPsA compared to NRD (both p < 0.0001), with no difference between early and established disease. This suggests that anti-CD74 elevation is present early and remains stable over the disease course. ROC curve analysis demonstrated good discrimination between PsA and NRD (AUC 0.80, p < 0.0001). A threshold of >15 U/mL yielded high specificity (98%) but limited sensitivity (41%), corresponding to a positive likelihood ratio (LR+) of 18 and a negative likelihood ratio (LR−) of 0.6. These characteristics indicate that anti-CD74 is better suited as a rule-in biomarker rather than a screening tool.

**Figure 1:**
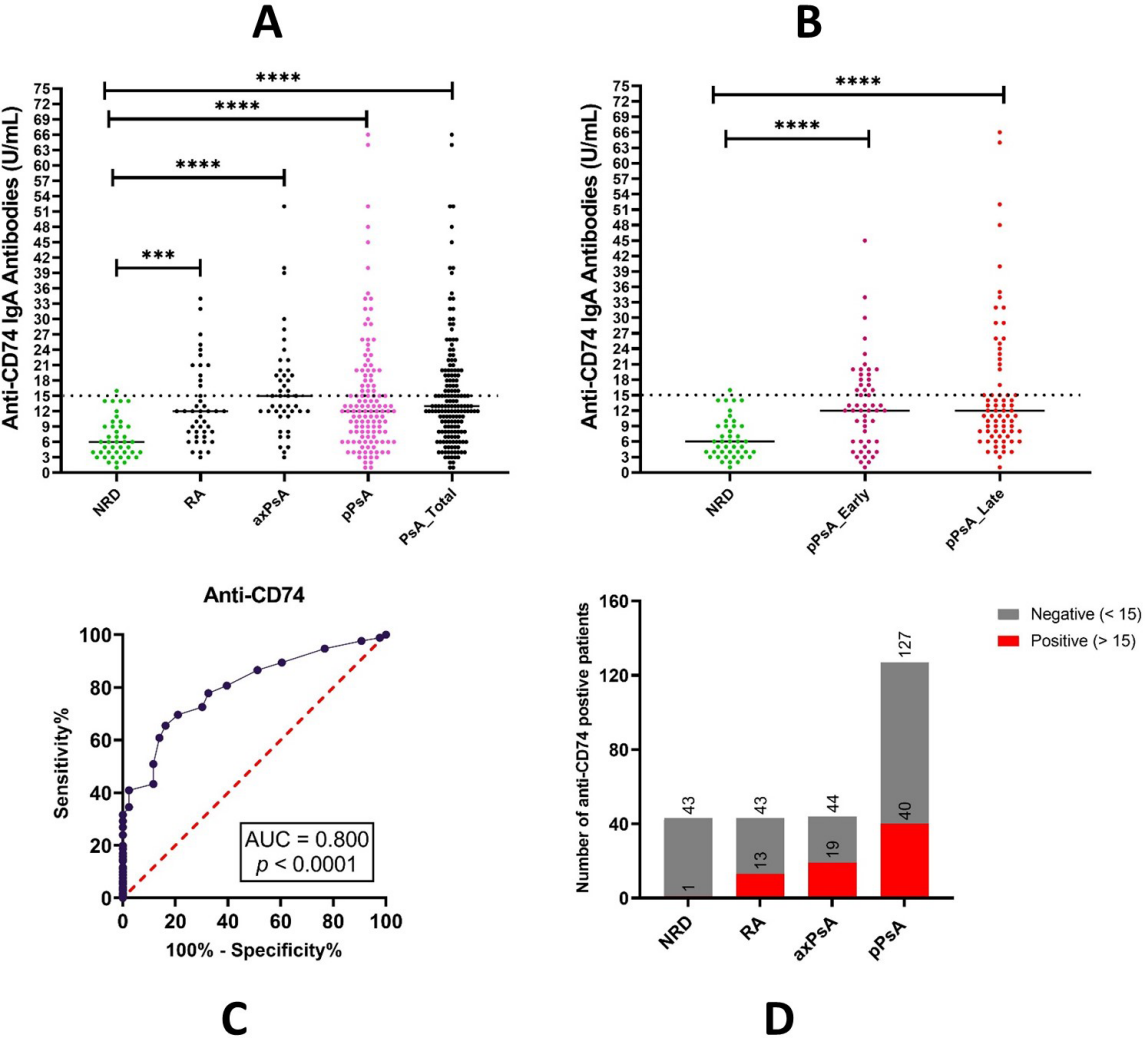
IgA anti-CD74 antibody levels and positivity rates in PsA patients and control groups. **(A)** Serum levels of IgA anti-CD74 antibodies were tested in NRD (n = 43), rheumatoid arthritis patients (RA, n = 43), total PsA (n = 171), pPsA (n = 127), and axPsA (n = 44). RA was included as an inflammatory disease control group to assess disease specificity. **(B)** Serum IgA anti-CD74 antibodies were tested in pPsA subgroups stratified by disease duration: ≤1 year (n = 53) and >1 year (n = 74). Each dot represents one sample. Horizontal lines represent median values. The dotted line represents the positive limit of >15 U/mL. Statistical comparisons were made using the Kruskal-Wallis test, followed by Dunn’s *post-hoc* test. Fisher’s exact test was used to compare the positive rates of pPsA and axPsA to those of the NRD group. **** Adjusted p-value < 0.0001. **(C)** ROC curve analysis for IgA anti-CD74 antibodies showed an AUC of 0.8 and p < 0.0001. **(D)** Anti-CD74-positive individuals (cutoff ≥ 15 U/mL) in NRD (1/43), RA (13/43), pPsA (40/127), and axPsA (19/44). *** indicates p < 0.001, **** indicates p < 0.0001.

Using the threshold of > 15 U/ml, anti-CD74 positivity was observed in 31.5% of pPsA and 43.2% of axPsA patients, compared with 2.3% in NRD controls (p < 0.0001; **Figure 1**). In the RA cohort, positivity was 30.2%, further supporting the notion that anti-CD74 reflects inflammatory disease activity rather than disease-specific processes.

### 3.3 Anti-CD74 and treatment escalation in pPsA

To evaluate whether anti-CD74 levels are associated with treatment escalation, analyses were first performed in the pPsA subgroup, followed by supportive analyses in the axPsA subgroup.

#### 3.3.1 pPsA subgroup

In pPsA, anti-CD74 levels showed a stepwise increase across treatment categories, with the lowest levels observed in untreated patients and higher levels in patients receiving csDMARDs and bDMARDs.

**Figure 2:**
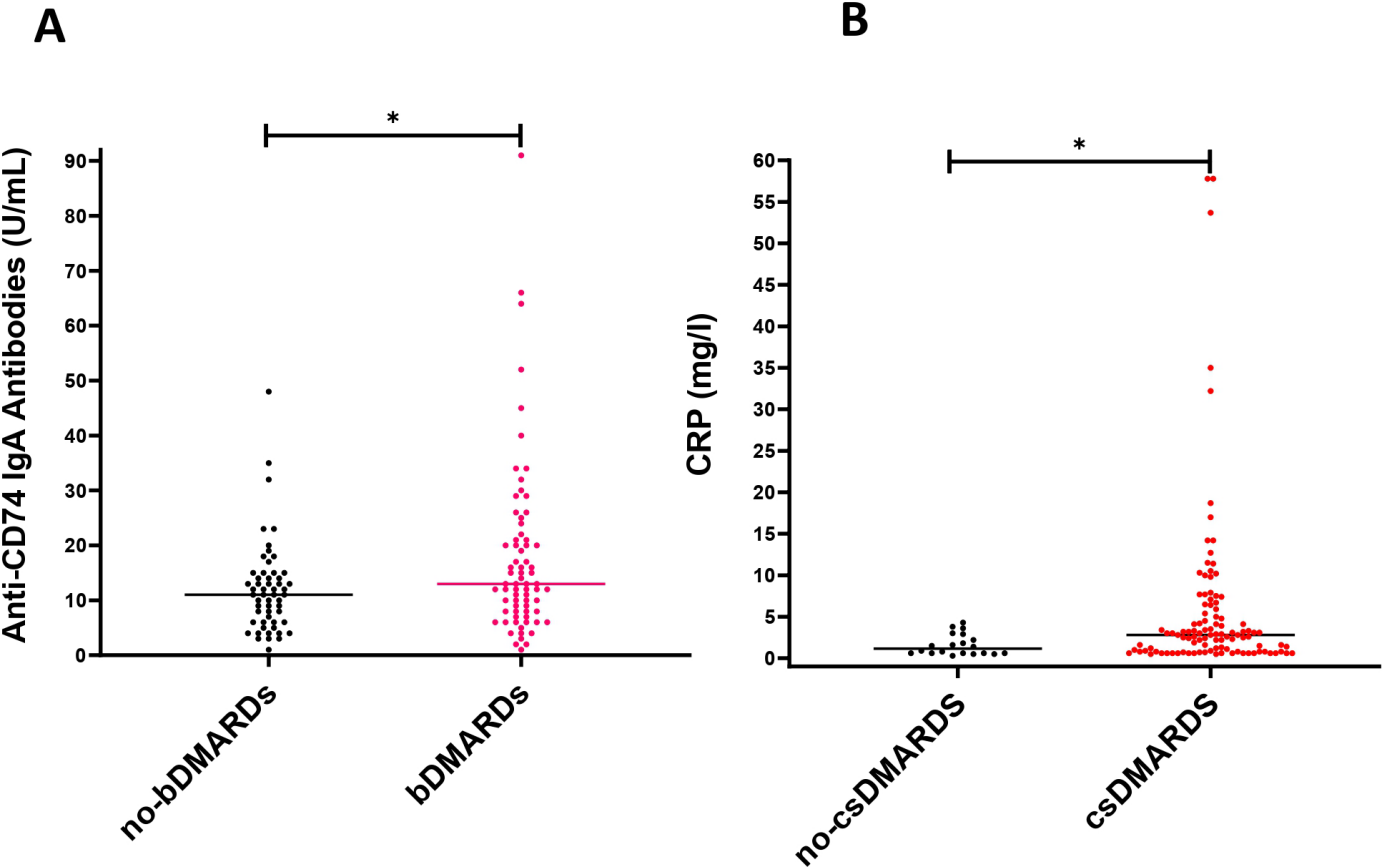
CD74 autoantibody and CRP levels in pPsA patients stratified by treatment. **(A)** Anti-CD74 levels in patients receiving bDMARDs (n = 71) and those not receiving bDMARDs (n = 55); p = 0.04. **(B)** CRP levels in patients receiving csDMARDs (n = 106) and those not receiving csDMARDs (n = 20); p = 0.003. Mann–Whitney U test was used for statistical comparisons. Lines represent medians. Statistical significance was considered at p < 0.05. * indicates p < 0.05.

When analyzed by treatment class (data were available for 126 patients), anti-CD74 levels were numerically higher in csDMARD-treated patients (n = 106) compared to untreated individuals (n = 20) (median 12.5 vs 10.5 U/mL); however, this difference did not reach statistical significance (p = 0.053). In contrast, patients receiving bDMARDs (n = 71) had higher anti-CD74 levels than those not receiving biologics (n = 55) (median 13.0 vs. 11.0 U/mL; p = 0.04) (**Figure 2**).

CRP levels were significantly higher in csDMARD-treated patients compared to untreated individuals (median 2.8 vs 1.15 mg/L; p = 0.003), whereas no significant difference was observed between bDMARD users and non-users (p = 0.09).

#### 3.3.2 axPsA subgroup

In axPsA, anti-CD74 levels were numerically higher in treated patients compared to untreated individuals; however, no statistically significant differences were observed for either csDMARD use (p = 0.7) or bDMARD use (p = 0.08). Similarly, CRP levels did not differ significantly between treated and untreated groups (all p > 0.3).

### 3.4 Relationship of anti-CD74 to clinical and immunological variables

To contextualize the role of anti-CD74 within established clinical and immunological frameworks, its association with demographic, inflammatory, and serological variables was evaluated.

In the pPsA subgroup, anti-CD74 levels showed no association with age (r = 0.128, p = 0.15) or CRP (r = −0.014, p = 0.9), indicating that anti-CD74 is not directly linked to conventional markers of systemic inflammation or to demographic factors such as age.

**Figure 3:**
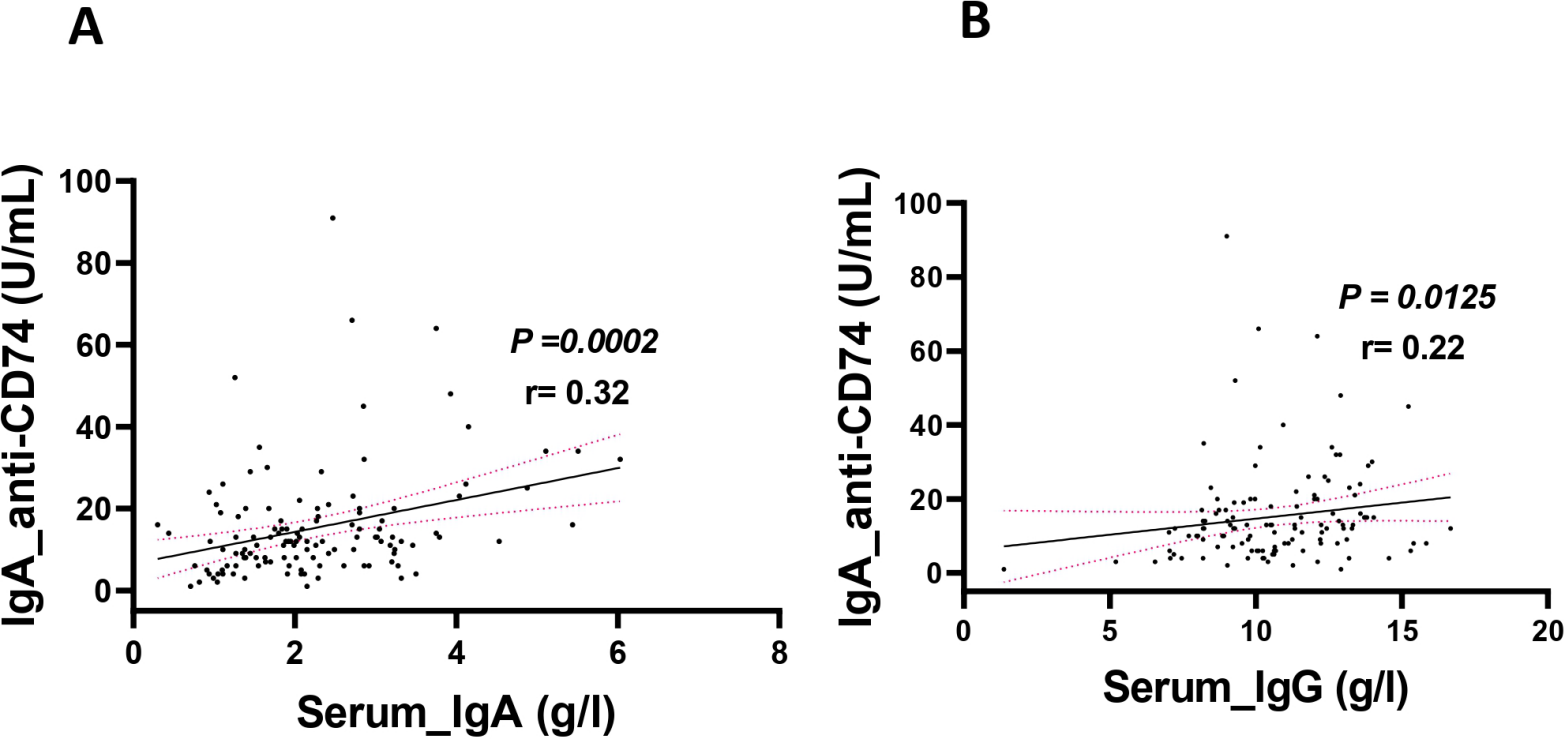
Correlation of CD74 autoantibody levels with IgA and IgG in pPsA patients. **(A)** CD74 autoantibody levels showed a moderate positive correlation with serum IgA (r = 0.32, 95% CI: 0.15 to 0.48, p = 0.0002; n = 125). **(B)** There was a weaker but still statistically significant correlation between CD74 and serum IgG levels (r = 0.22, 95% CI: 0.044 to 0.39, p = 0.0125; n = 125). Spearman’s rank correlation test was used due to the non-normal distribution of the variables. Statistical significance was considered at p < 0.05.

In contrast, anti-CD74 levels demonstrated a moderate positive correlation with total IgA (r = 0.32, p = 0.0002) and a weaker correlation with IgG (r = 0.22, p = 0.0125). After false discovery rate (FDR) correction, both associations remained significant (IgA: q = 0.0008; IgG: q = 0.0250), with a stronger correlation coefficient observed for IgA, indicating a more pronounced relationship. This pattern may be consistent with the known role of IgA-dominated responses in mucosal and barrier-associated immunity, although no mechanistic conclusions can be drawn from the present data (**Figure 3**).

In the axPsA subgroup, no significant correlations were observed between anti-CD74 and age, CRP, IgA, or IgG.

### 3.5 Independent association of anti-CD74 with treatment escalation

To determine whether the association between anti-CD74 and treatment escalation is independent of established clinical and inflammatory variables, univariate and multivariate logistic regression analyses were performed. The outcomes of interest were the use of csDMARD and bDMARD therapies in patients with pPsA. Independent variables included CD74 autoantibody levels, age, sex, CRP, HLA-B27, current smoking status, arthritis, enthesitis, dactylitis, and serum IgA levels.

Univariate analysis in the pPsA subgroup revealed that age (OR: 1.05, 95% CI: 1.006–1.09, p = 0.032) and CRP (OR: 1.46, 95% CI: 1.114–2.14, p = 0.024) were significantly associated with csDMARD use, whereas anti-CD74 levels were not. However, in the multivariate model adjusting for clinical and serological variables, anti-CD74 levels were independently associated with csDMARD use (OR: 1.113, 95% CI: 1.026–1.23, p = 0.022) (**Table 2**). The model demonstrated good discrimination (AUC 0.81, 95% CI: 0.71–0.91).

**Table 2:** Univariate and multivariate logistic regression analysis predicting csDMARD therapy use in the pPsA patients.

Variable	Regression models for csDMARD use					
	Univariate models				Multivariate models	
	OR (95% CI)	*p-*value	AUC	AUC *(p-*value)	OR (95% CI)	*p-*value
CD74	1.06 (1.005 to 1.2)	0.07	0.637	0.053	1.113 (1.026 to 1.23)	0.022*
Age	1.05 (1.006 to1.09)	0.032*	0.657	0.0267	1.03 (0.9944 to 1.08)	0.17
CRP	1.46 (1.114 to 2.14)	0.024*	0.708	0.003	1.37 (1.037 to 2.104)	0.09
Smoking	2.02 (0.355 to 38.18)	0.51	0.523	0.74	2.49 (0.33 to 55.34.)	0.45
Arthritis	2.4 (0.60 to 8.19)	0.18	0.553	0.45	3.02 (0.6 to 14.41)	0.16
Enthesitis	1.60 (0.60 to 4.54)	0.36	0.556	0.43	1.47 (0.46 to 4.89)	0.52
Dactylitis	0.77 (0.29 to 2.2)	0.61	0.529	0.68	0.76 (0.23 to 2.59)	0.65
Male	1.024 (0.39to 2.8)	0.96	0.503	0.97	0.96 (0.31 to 3.07)	0.95
Serum IgA	0.83 (0.55 to 1.28)	0.38	0.577	0.28	0.58 (0.32 to 1.01)	0.06
HLA-B27	0.34 (0.078 to 1.8)	0.17	0.564	0.45		

*p-value was significant if <0.05. Bold values indicate statistically significant associations (p < 0.05).

For bDMARD therapy, anti-CD74 levels were significantly associated with treatment in both univariate (OR: 1.043, 95% CI: 1.009–1.088, p = 0.03) and multivariate analyses (OR: 1.052, 95% CI: 1.013–1.103, p = 0.02) (**Table 3**), while no other variables showed consistent associations. The corresponding multivariate model showed moderate discriminative performance (AUC 0.67, 95% CI: 0.57–0.76, p = 0.002).

**Table 3:** Univariate and multivariate logistic regression analysis predicting bDMARD therapy use in the pPsA patients.

Variable	Regression models for subsequent bDMARD therapy					
	Univariate models				Multivariate models	
	OR (95% CI)	*p-*value	AUC	AUC *(p-*value)	OR (95% CI)	*p-*value
Anti-CD74	1.043 (1.009 to 1.088)	0.03*	0.607	0.04*	1.052 (1.013to1.103)	0.02*
Age	0.99 (0.97 to 1.018)	0.65	0.555	0.29	0.99 (0.97 to 1.02)	0.62
CRP	0.99 (0.96 to1.039)	0.93	0.589	0.09	0.99 (0.95 to 1.04)	0.62
Smoking	0.62 (0.17 to 2.16)	0.44	0.519	0.71	0.71 (0.18 to 2.7)	0.61
Arthritis	2.58 (0.84 to 8.87)	0.11	0.547	0.37	2.8 (0.83 to 10.8)	0.11
Enthesitis	1.06 (0.52 to 2.16)	0.87	0.507	0.89	1.023 (0.50 to 2.24)	0.95
Dactylitis	0.81 (0.37 to 1.74)	0.58	0.523	0.66	0.82 (0.36 to 1.86)	0.63
Male	1.36 (0.66 to 2.82)	0.41	0.537	0.48	1.24 (0.57 to 2.7)	0.59
Serum IgA	1.017 (0.73 to 1.42)	0.92	0.507	0.89	0.81 (0.54 to 1.19)	0.28
HLA-B27	0.74 (0.17 to 2.99)	0.67	0.515	0.82		

*p-value was significant if <0.05. Bold values indicate statistically significant associations (p < 0.05).

In contrast, in the axPsA subgroup, no consistent associations between anti-CD74 and csDMARD use were observed. For bDMARD therapy, anti-CD74 reached significance only in the multivariate model (OR: 1.35, 95% CI: 1.08–1.90, p = 0.032), alongside age and smoking status, suggesting a less robust and potentially heterogeneous relationship in axial disease in our cohort.

### 3.6 Longitudinal analysis in early pPsA

To further explore whether baseline anti-CD74 levels are associated with subsequent treatment decisions over time, a prospective analysis was conducted in the early pPsA subgroup (disease duration ≤ 1 year).

During follow-up, patients who initiated csDMARD therapy (n = 43) showed higher baseline anti-CD74 levels than those who remained untreated (n = 10) [median 13.0 U/mL (IQR 9.0–19) vs 6.0 U/mL (IQR 3.75–12.3); p = 0.047]. However, after FDR correction, this association did not remain statistically significant (q = 0.094), indicating that this finding should be interpreted cautiously. The majority of patients began csDMARD medication during the first year, with just one patient starting beyond two years.

In contrast, baseline anti-CD74 levels were not significantly different between patients who later initiated bDMARD therapy (n = 28) and those who did not (n = 25) [median 13.0 U/mL (IQR 9.25–20.0) vs. 12.0 U/mL (IQR 4.5–16.0); p = 0.19; q = 0.1866]. The majority of bDMARD therapy was initiated between 1 and 2 years, with 1 patient starting treatment within 4 years.

Overall, these findings provide limited longitudinal support for an association between anti-CD74 and treatment escalation, with no statistically significant associations remaining after correction for multiple testing. Given the small sample size and exploratory nature of this analysis, these results should be interpreted cautiously and considered hypothesis-generating.

### 3.7 Exploratory clinical modifiers: BMI and psoriasis outcomes

To explore potential clinical modifiers of anti-CD74 levels and treatment patterns, additional analyses were performed in the early pPsA subgroup (n = 51).

Body mass index (BMI) showed a broad distribution, with 31.4% of patients classified as obese (BMI ≥ 30 kg/m²) and 33.3% as overweight (23), consistent with the known metabolic burden in PsA (**Table 4**). When stratified by median BMI (27.66 kg/m²), no significant difference in anti-CD74 levels was observed between the lower (n = 26) and higher (n = 26) BMI groups (median 12.0 vs 15.0 U/mL; p = 0.68), indicating that anti-CD74 is not directly influenced by body mass.

**Table 4:** Clinical characteristics and psoriasis outcomes in early pPsA patients (n = 51).

Variable	Category	n (%)
BMI classification	Normal weight (<25 kg/m²)	18 (35.3%)
	Overweight (25–29.9 kg/m²)	17 (33.3%)
	Obesity (≥30 kg/m²)	16 (31.4%)
Psoriasis outcome during follow-up	Complete resolution	5 (9.8%)
	Mild activity/improvement	5 (9.8%)
	Active disease	18 (35.3%)
	Not available (NA)	23 (45.1%)

BMI was calculated depending on the available height and weight data. BMI categories were defined according to WHO standards: normal weight (<25 kg/m²), overweight (25–29.9 kg/m²), and obese (≥30 kg/m²) (23). Psoriasis outcomes during follow-up were classified as complete resolution, mild activity/improvement, or active disease.

BMI was further analyzed in relation to treatment patterns. A nominal association was observed between higher BMI and csDMARD use (24/25 vs 18/26; p = 0.024); however, this association did not remain statistically significant after FDR correction (q = 0.0714). Similarly, no association was observed between BMI and bDMARD use (p > 0.999; q = 0.9999).

Psoriasis outcomes following methotrexate therapy were heterogeneous. Among patients with available follow-up data, complete clearance was observed in a minority (9.8%), while the majority exhibited either persistent disease activity (35.3%) or partial improvement (9.8%), reflecting variability in treatment response.

### 3.8 RA subgroup analysis

To further contextualize the immunological relevance of anti-CD74, analyses were performed in the RA subgroup.

No significant correlations were observed between anti-CD74 levels and established serological markers, including RF (r = −0.12, p = 0.43) and ACPA (r = −0.047, p = 0.76), nor with CRP levels (r = −0.0038, p = 0.98).

These findings indicate that, in RA, anti-CD74 is not associated with classical autoantibody profiles or systemic inflammatory markers, supporting the notion that its immunological associations differ across inflammatory rheumatic diseases.

## 4 Discussion

PsA is a heterogeneous inflammatory disease within the SpA spectrum, characterized by a combination of peripheral manifestations including arthritis, enthesitis, and dactylitis, and, in a subset of patients, axial involvement. Despite its substantial disease burden, PsA remains diagnostically and clinically challenging, particularly in early disease stages, where heterogeneous presentation may contribute to delayed diagnosis (24–30). This is clinically relevant, as early initiation of effective therapy has been shown to improve long-term outcomes (31). In this context, identifying mechanistically informative biomarkers that reflect underlying disease processes and support treatment decision-making represents a major unmet need in PsA. In line with this objective, the present study was structured to evaluate anti-CD74 primarily in relation to treatment escalation, with secondary and exploratory analyses to contextualize this association within clinical and immunological frameworks.

In the existing literature, numerous investigations have shown that anti-CD74 IgA antibodies are increased in axSpA (7–12) and have high diagnostic specificity (10). Further research indicated that IgA anti-CD74 antibodies had 67% sensitivity and 95% specificity, with 85.1% of SpA patients testing positive, whereas only 5% of non-SpA patients tested positive (32). A separate study demonstrated that anti-CD74 IgA antibodies had the highest sensitivity and specificity in identifying young men with axSpA (11). A longitudinal investigation revealed markedly elevated plasma IgA anti-CD74 levels in radiographic axSpA patients compared with controls, and a correlation between baseline IgA anti-CD74 and subsequent radiographic spine changes (12). These results indicate that IgA anti-CD74 antibodies may function as possible biomarkers for the diagnosis of axSpA. Nevertheless, data on their function in pSpA, especially regarding early versus late stages of the illness, remain unclear.

A recent Egyptian case-control study, consistent with our findings, indicated elevated frequencies of IgA anti-CD74 positivity in axSpA (73.3%) and pPsA (66.6%), contrasted with a minimal prevalence in healthy controls (3.3%), highlighting that CD74 autoimmunity may encompass various SpA phenotypes rather than being confined to axial disease (13). Our investigation revealed markedly elevated IgA anti-CD74 levels in PsA compared with NRD controls, with no significant difference between pPsA and axPsA, suggesting that anti-CD74 is associated with inflammatory disease states rather than specific clinical phenotypes within PsA.

Notably, by including a disease-specific inflammatory control group (RA), we found that IgA anti-CD74 levels were similarly elevated in both RA and PsA, with no significant difference between the groups. This finding suggests that anti-CD74 is not disease-specific but instead reflects shared immunological processes across inflammatory rheumatic diseases. In this context, anti-CD74 may be more accurately regarded as an indicator of inflammatory disease burden rather than a diagnostic discriminator among specific conditions. In the RA subgroup, anti-CD74 IgA levels showed no association with RF, ACPA, or CRP, further supporting the concept that anti-CD74 reflects immunological pathways distinct from classical autoantibody-mediated or acute-phase inflammatory responses.

When categorized by illness duration, both early and late pPsA patients had substantially elevated IgA anti-CD74 levels compared to NRD, with no significant difference between disease stages. These findings suggest that anti-CD74 elevation occurs early and persists over time, supporting its potential role as a stable immunological feature rather than a marker of cumulative disease damage. While prior studies in axSpA have reported conflicting results regarding temporal dynamics (7, 8, 33, 34), our data support a model in which anti-CD74 represents a sustained component of disease-associated immune activation in pPsA. Within this framework, the primary finding of this study was the association between anti-CD74 and treatment escalation. Anti-CD74 levels were higher in patients receiving bDMARD therapy, while differences for csDMARD use were weaker and did not consistently reach statistical significance in unadjusted analyses. Multivariate regression analysis further demonstrated that anti-CD74 was independently associated with both csDMARD and bDMARD use after adjustment for relevant clinical variables. These findings should be interpreted as reflecting associations with treatment patterns rather than direct predictive effects, given the observational design.

Importantly, these associations were observed despite comparable CRP levels across treatment groups, suggesting that anti-CD74 may capture immunological features not reflected by conventional inflammatory markers.

To further contextualize this finding within the clinical phenotype, additional disease domains were examined within the regression framework. Anti-CD74 levels were not associated with peripheral manifestations such as enthesitis or dactylitis, suggesting that the observed association with treatment escalation is unlikely to be driven solely by the extent of peripheral musculoskeletal involvement. These findings may reflect the biological independence of CD74-related immune responses from specific clinical domains, although limited statistical power cannot be excluded and warrants further investigation in larger cohorts.

These results partially align with previous findings in axSpA, where anti-CD74 has been associated with biologic therapy use (22), and extend this observation to a pPsA cohort. Taken together, the consistent pattern across analyses, the elevation in inflammatory disease, independence from CRP, and the association with treatment escalation support the concept that anti-CD74 reflects an immunological dimension of disease burden that may influence clinical decision-making. However, this interpretation remains associative and requires validation in prospective and mechanistic studies.

To further contextualize the biomarker, we evaluated its relationship with clinical and immunological variables. Anti-CD74 levels were not associated with age or CRP, indicating independence from conventional demographic and inflammatory parameters. In contrast, a moderate positive correlation was observed with total IgA (r = 0.32, p = 0.0002), and a weaker correlation with IgG (r = 0.22, p = 0.0125). Importantly, both associations remained statistically significant after FDR correction (IgA: q = 0.0008; IgG: q = 0.0250), with a stronger correlation coefficient observed for IgA, indicating a more pronounced relationship. These findings suggest that anti-CD74 is more closely linked to humoral immune activation, particularly IgA-related responses, rather than systemic inflammation as reflected by CRP. This interpretation is consistent with previous reports suggesting a role for mucosal immune activation in SpA pathogenesis (34–36), although this link remains incompletely understood. This interpretation is also supported by previous studies reporting no clear association between anti-CD74 and CRP or disease activity measures (13), repeated predominance of the IgA isotype in anti-CD74 responses (34, 35), and evidence linking CD74 expression to intestinal immune pathways (36), although contradictory data indicate that this mucosal link remains unproven (21). The weaker but still significant association with IgG may reflect broader humoral immune activation, including ongoing antigenic stimulation and polyclonal B-cell activation in chronic inflammatory conditions (18, 37, 38).

Exploratory analyses in the early pPsA subgroup provided additional clinical context. Baseline anti-CD74 levels were higher in patients initiating csDMARD therapy during follow-up (p = 0.047); however, this association did not remain statistically significant after false discovery rate (FDR) correction (q = 0.094), indicating that this finding should be interpreted cautiously. No statistically significant association was observed for bDMARD initiation (p = 0.19; q = 0.1866). Given the limited sample size and multiple testing within this exploratory framework, these findings should be considered hypothesis-generating.

Similarly, no association was observed between anti-CD74 and BMI, suggesting that the biomarker is not directly influenced by metabolic status. Although BMI showed a nominal association with csDMARD use, this did not remain significant after FDR correction, indicating that this observation may reflect chance rather than a robust relationship.

Psoriasis outcomes following methotrexate therapy were heterogeneous, with only a minority achieving complete clearance. These findings were descriptive and not associated with anti-CD74 levels, consistent with their classification as exploratory analyses. Nonetheless, they underscore the clinical heterogeneity of early PsA and the need for biomarkers that may help stratify patients according to disease burden and treatment response.

This study has several strengths. In line with EULAR recommendations for biomarker research, we combined cross-sectional and prospective analyses in a well-characterized pPsA cohort, allowing assessment of both disease-associated antibody levels and their relationship to subsequent treatment decisions. This design enabled us to position anti-CD74 within a clinically relevant framework, linking immunological findings to treatment escalation while maintaining a predefined analytical hierarchy.

Nevertheless, several limitations should be considered. The study is primarily observational, precluding causal inference, and the reported associations should be interpreted as reflecting treatment patterns rather than predictive effects. Although a prospective component was included, anti-CD74 was measured at a single time point, limiting the assessment of longitudinal biomarker dynamics and treatment-related changes. The study population was derived from a single center, which may limit generalizability. In addition, the comparator group consisted of patients with non-inflammatory musculoskeletal conditions rather than healthy individuals or multiple disease-specific control groups, restricting the ability to assess disease specificity.

The sample sizes for the subgroup and longitudinal analyses were limited, particularly for treatment initiation and exploratory endpoints, reducing statistical power and increasing susceptibility to variability. Accordingly, borderline or non-significant findings were interpreted with caution and framed as hypothesis-generating. Although false discovery rate (FDR) correction was applied to predefined families of exploratory analyses, a residual risk of type I error cannot be excluded given the number of comparisons performed.

Treatment exposure was categorized into csDMARD and bDMARD groups as a pragmatic representation of major therapeutic strategies in clinical practice; however, this approach does not capture the full complexity of treatment pathways, including combination regimens, treatment sequencing, or drug-specific effects. Moreover, treatment outcomes were limited to initiation and escalation, and we were unable to assess treatment discontinuation or response, including bDMARD stoppage due to inefficacy or remission. These outcomes are clinically highly relevant, as they may provide insight into whether anti-CD74 is associated not only with treatment escalation but also with treatment persistence and effectiveness.

Finally, exploratory analyses involving BMI, psoriasis outcomes, and specific clinical domains were limited by sample size and were not adjusted in multivariable models; they should be considered descriptive. The absence of association between anti-CD74 and certain clinical features, such as enthesitis or dactylitis, may reflect either biological independence or insufficient statistical power. Future studies in larger, multicenter cohorts with serial biomarker measurements and detailed treatment outcome data will be essential to validate these findings and further define the clinical utility of anti-CD74 in PsA.

## 5 Conclusion

In conclusion, IgA anti-CD74 antibodies were elevated in PsA and remained relatively stable across disease duration. In pPsA, higher anti-CD74 levels were associated with treatment escalation and were independent of conventional inflammatory markers such as CRP, suggesting that this biomarker may reflect a distinct immunological component of disease burden. However, several secondary and exploratory findings require cautious interpretation, particularly in view of the observational design, subgroup size, and multiple-testing considerations. Therefore, while anti-CD74 appears to be a promising biomarker candidate for disease stratification in pPsA, its clinical utility requires confirmation in larger, multicenter longitudinal studies with serial biomarker assessment and standardized treatment outcome.

## Data Availability

Data in this study are presented in the main text. Please contact the corresponding author for further inquiries.
