# Correction: IgA anti-CD74 autoantibodies are associated with treatment escalation in peripheral psoriatic arthritis

**DOI:** 10.3389/fimmu.2026.1887365

**Published:** 2026-05-27

**Authors:** Alaa Elsaghir, Luise Kuske, Katja Kniesch, Miriam Rabenow, Till Strowig, Torsten Witte

**Affiliations:** 1 Department of Rheumatology and Immunology, Hannover Medical School, Hannover, Germany; 2 Department of Microbiology & Immunology, Faculty of Pharmacy, Assiut University, Assiut, Egypt; 3 Helmholtz Centre for Infection Research, Braunschweig, Germany; 4 Center for Individualized Infection Medicine, a joint venture of Hannover Medical School and the Helmholtz Center for Infection Research, Hannover, Germany

**Keywords:** psoriatic arthritis, peripheral psoriatic arthritis, IgA anti-CD74, biomarker, treatment escalation, bDMARDs, csDMARDs

The wrong reference citation was used in section **4 Discussion**, paragraph 9. Instead of reference 37, it should have cited reference 36. The corrected sentence reads:

“This interpretation is also supported by previous studies reporting no clear association between anti-CD74 and CRP or disease activity measures (13), repeated predominance of the IgA isotype in anti-CD74 responses (34, 35), and evidence linking CD74 expression to intestinal immune pathways (36), although contradictory data indicate that this mucosal link remains unproven (21).”

Additionally, the wrong reference citation was used in the footnote for **Table 4**. Instead of reference 38, it should have cited reference 23. The corrected caption of **Table 4** appears below:

“BMI was calculated depending on the available height and weight data. BMI categories were defined according to WHO standards: normal weight (<25 kg/m²), overweight (25–29.9 kg/m²), and obese (≥30 kg/m²) (23). Psoriasis outcomes during follow-up were classified as complete resolution, mild activity/improvement, or active disease.”

The original version of this article has been updated.

